# Scleritis in rheumatoid arthritis: Before and during biologic era

**DOI:** 10.3389/fopht.2023.1106419

**Published:** 2023-01-31

**Authors:** Daphne P. C. Vergouwen, Pascal H. P. de Jong, Marco W. J. Schreurs, Josianne C. Ten Berge, Aniki Rothova

**Affiliations:** ^1^ Department of Ophthalmology, Erasmus MC, University Medical Center, Rotterdam, Netherlands; ^2^ Department of Immunology, Erasmus MC, University Medical Center, Rotterdam, Netherlands; ^3^ Department of Rheumatology, Erasmus MC, University Medical Center, Rotterdam, Netherlands

**Keywords:** scleritis, rheumatoid arthritis, incidence, prevalence, rheumatoid factor (RF), anti-citrullinated protein antibodies (ACPAs)

## Abstract

**Objectives:**

Scleritis represents a severe extra-articular manifestation of rheumatoid arthritis (RA). Recent clinical observations suggest a decreasing incidence of scleritis in RA, attributed to improved treatment options. Our study reports on the incidence and clinical characteristics of scleritis in RA observed in the biological era and reflects on our results in a historical perspective.

**Methods:**

We performed a retrospective evaluation of all 1623 consecutive patients with RA diagnosed at the department of rheumatology between 2011 and 2021 at the Erasmus Medical Center to investigate the incidence of scleritis. We also reviewed clinical and laboratory data of all patients with scleritis and RA from the department of ophthalmology at our center. In addition, we reviewed the literature on this topic and discuss our results in view of changes over time.

**Results:**

The incidence of scleritis within recent series of patients with a diagnosis of RA in our tertiary center was 0,25% in 10 years (4 out of 1623; 2011-2021).The cumulative incidence of scleritis in RA based on literature review from the pre-biologic era varied from 0.7% per 8 years to 0,8% per 30 years. Manifestations and complications of scleritis remained unchanged over time, with scleral necrosis developing in more than 80% of cases and mortality of RA patients with scleritis remained similar to pre-biologic era (30% in 9 years after the onset of scleritis). The RA patients with scleritis often exhibited autoantibodies (rheumatoid factor and/or anti-citrullinated protein antibody) and erosive disease.

**Conclusion:**

Although our recent series is characterized by a slightly lower incidence of scleritis in RA compared to the pre-biologic era, clinical presentation remained severe and similar to the pre-biologic era. Ophthalmologists and rheumatologists should be aware of scleritis as a severe extra-articular manifestation of RA.

## Introduction

Scleritis is a severe and painful ocular disorder, commonly associated with complications that often lead to visual loss and decreased quality of life (1, 2). Approximately half of the cases is associated with a systemic autoimmune mediated diseases, of which rheumatoid arthritis (RA) forms the majority (3). RA classically causes a symmetrical polyarthritis of the small joints (4). The disease may also affect skin, eyes, lungs, heart or nerves, and these extra-articular manifestations considerably increase the burden of disease (5, 6).

Eye involvement in RA usually manifests as scleritis, which is characterized by multiple complications and increased mortality (7). Previous literature showed that scleritis occurred more often in RA patients with autoantibody positivity, including rheumatoid factor (RF) and/or anti-citrullinated protein antibody (ACPA), and erosive disease (8, 9). Most studies on scleritis and RA originate from the pre-biologic era (before the year 2000). In the late 1990’s the first biological agents, such as TNFα-inhibitors became available, which were highly effective in RA. Since the introduction of these biologics the incidence of scleritis in RA might be decreasing according to recent clinical observations (10). However, the exact incidence and severity of scleritis in RA from the current era of modern treatment strategies is not known.

We performed a retrospective cohort study and reviewed the literature on prevalence of scleritis in RA to investigate whether the incidence of scleritis in RA has changed over time as a result of improved treatment options. We also examined the clinical and serological characteristics of recent scleritis cases in patients with RA, who were diagnosed in our tertiary care center.

## Methods

We performed a retrospective study of all 1623 consecutive patients diagnosed with RA at the department of rheumatology at the Erasmus MC (University Medical Center, Rotterdam, The Netherlands) between 2011 and 2021. The medical files were reviewed for the terms ‘scleritis’ or ‘sclerouveitis’ to asses the incidence of scleritis in RA.

An ophthalmological assessment of all patients with scleritis and RA, who consulted the department of ophthalmology from 2011 to 2021 was also performed (includes also patients treated by rheumatologists from other centers). All had an ophthalmological follow-up of at least 9 months. Included were solely patients with non-infectious scleritis. Collected data included patient demographics, clinical manifestations, complications, and treatment regimen for both scleritis and RA. In addition, diverse laboratory data on the presence of serum autoantibodies, including RF, ACPA and the more recently identified anti-RA33 antibody, during an arbitrary moment in the disease course was reviewed (11). RF (IgA and IgM), ACPA (IgA and IgG), and anti-RA33 (IgA, IgG and IgM) were determined using fluorescence enzyme immunoassay (FEIA) on the Phadia 250 system according to manufacturer’s instructions (Thermo Fisher Scientific, Freiburg, Germany). Whenever possible, missing laboratory data were supplemented from frozen stored sera of included patients.

We reviewed the available literature to evaluate the prevalence and possible risk factors of scleritis in RA. The PubMed database was searched using the terms ‘Scleritis’ and ‘Rheumatoid arthritis’ for all accessible studies up to August 2021. We included studies that fulfilled the following criteria: full text available in English, cohort studies with data on incidence/prevalence, or data on clinical characteristics and complications of scleritis in RA. If available, serological characteristics were also included. Out of 232 available studies, we included 7 studies with data on incidence and/or prevalence as well as reporting on clinical characteristics. Biologicals became commonly available in our country from the early 2000’s, therefore our study was considered to take place in a biologic era. Pre-biologic era was considered for studies in which biological drugs were not yet available.

Continuous data were reported as mean ± range or standard deviation (SD), and categorical data were reported as number with percentage. The local Medical Ethics Committee (Erasmus MC, MEC-2012-016) has reviewed and approved this study, and waived requirement for informed consent. The research was performed according to the Tenets of the Declaration of Helsinki.

## Results

### Incidence and prevalence of scleritis

Out of the 1623 consecutive patients with RA, 4 developed scleritis, resulting in a 10-year cumulative incidence of 0,25%. Two additional patients had scleritis, and 1 sclero-uveitis before our study period started and, therefore, the prevalence of scleritis in RA was 0,43% (7/1623; **Table 1**).

**Table 1:** Incidence or prevalence of scleritis within rheumatoid arthritis cohorts over time.

Study	Country	Year	Number RA patients	Study design	Patient selection	Scleritis, Number (%)	Outcome
Pre- biologic era							
McGavin	Scotland	1976	4210	Retrospective	Rheumatology	28 (0.67)	Incidence (1965-1973)
Turesson	USA	2003	609	Retrospective	Rheumatology	4 (0.8)	Incidence (1955-1994)
Carmona^a^	Spain	2003	788	Retrospective	Rheumatology	12 (1.5)	Incidence (1989-1999)
Biologic era							
Moura^a^	Brazil	2012	262	Retrospective	Rheumatology outpatient clinic	4 (3.5)	Prevalence (Search January-December 2010)
Berkenstock	USA	2021	19.923	Retrospective	Health system wide electronic record search ^C^	184 (0.9)	Prevalence (Search 2013- 2018)
Current study	The Netherlands	2021	1623	Retrospective	Rheumatology outpatient clinic	4^b^ (0,25) 7 (0,43)	Incidence (2011-2021) Prevalence (Search 2011-2021)

RA, Rheumatoid arthritis.

a Scleritis and episcleritis were taken together.

b 4 patients developed scleritis within the study period and 3 additional patients had scleritis already before the study started.

c attempts to bypass referral bias.

The large cohort studies that reported on the incidence/prevalence of scleritis in RA over time are shown in **Table 1**. McGavin et al. studied a large number of patients with RA (N=4210) and showed an 8-year cumulative incidence from 1965 of 0.67%. Turesson et al. studied 609 patients with RA, and estimated a cumulative incidence of 0.8% within a 30-year time period (research period 1955 - 1994) (12, 13). A comprehensive study by Berkenstock et al. noted a prevalence of 0.9% within a 5-year period (2013 – 2018). Noteworthy is the fact that all patients who ever had scleritis were included in this study (3).

### Clinical characteristics of scleritis patients

Next to the 7 patients with scleritis and RA who were treated by rheumatologists in our center, an additional 3 patients with scleritis and RA were seen in our ophthalmology department (these 3 additional patients were treated for their RA in other hospitals, which explains the disparity in numbers). Ocular complications and characteristics of our ophthalmological case series are shown in **Tables 2**, **3**. In 3 out of 10 patients (30%) scleritis developed before the diagnosis of RA was made. Scleritis preceded arthritis onset by 1, 7 and 13 years. In the other 7 patients the mean interval between the onset of RA and development of scleritis was 11.6 years. Complications of scleritis such as necrosis and visual loss remained common. Also, mortality of patients with scleritis and RA remained high (30%), all within 9 years after the onset of scleritis (**Table 3**). The majority of patients suffered from a recurrent or chronic course of scleritis and most of them had other extra-articular RA manifestations. The 7 patients with RA who developed scleritis during the disease course were all treated with DMARDs and 3 even with biologics prior to onset of scleritis (**Table 3**), however, this systemic medication was stopped in 3 out of 7 patients shortly before the onset of scleritis. The comparison of scleritis severity in patients with and without biologic drugs prior to onset of scleritis was not reliable due to the limited number of patients, however all 3 patients with prior biologics had severe scleritis complicated by scleral necrosis.

**Table 2:** Characteristics, risk factors and complications of scleritis in rheumatoid arthritis patients over time.

Study	Mean age (years ± SD or range)	Female (%)	Interval RA to scleritis(years)	Mean FU (years)	RA	Extra-articular manfestations	Laboratory parameters	Clinical features scleritis	Necrosis, (%)	Visual impairment (%)	Mortality (%)	Remarks
Jayson 1971 N=14	56.5 ± 10.7	57	13 1/14 scleritis first	-^a^	All erosive RA	71% RN, 17% pleurisy/pericarditis, 71% arteritis	High ESR, all Waaler-Rose titer >1:32^b^	-	-	n.s.	-	9/142 from RA cohort, 5 from other referrals
McGavin1976 N=37	58.3 ± 11.8	68	14.5^c^	Cross-sectional	High X-ray stage (more erosive RA)	50% RN, 9% peripheral neuropathy	High ESR, 23.5% smooth muscle antibodies	67.6% bilateral, 29.1% PS abnormalities	81	36.6% VA <6/9	45.5^d^	28/4210 from RA cohort, 9 from other referrals
Caimmi 2018 N=33	65 (52-71)	67	14.6	5.1	52% radiographic erosions	36% RN, 27% severe exRA	ESR & CRP normal 91% seropositive (RF or ACPA) 36% ANA	33% bilateral, 10% CME	51	65% visual loss (> 2 Schnellen lines)	15% 5y 30% 10y^e^	All from RA cohort
Current study 2021 N=10	59.7^f^ ± 11.9	80	11.6 3/10 scleritis first	6.5	50% erosive RA	56%^g^	89% RF positive, all ACPA positive	70% bilateral, 50% complications	90	50% lowest VA ≤20/40 10% ≤20/200 20% final VA ≤20/40	30%^h^	7 from RA cohort, 3 from ophthalmological referrals

RA, Rheumatoid arthritis; FU, Follow up; PS, posterior segment; CME, Cystoid macular edema; ExRA, Extra-articular manifestations of RA; RN, Rheumatoid noduli; ESR: Erythrocyte sedimentation rate; CRP, C-reactive protein; VA, visual acuity; RF, rheumatoid factor; ANA, Antinuclear antibodies; ACPA, anti-citrullinated peptide antibodies n.s., not specified.

a All empty cells: result not given in this study.

b Waaler-rose titer: agglutination test, previously used method to detect rheumatoid factor.

c Time onset RA to episcleritis/scleritis.

d By 1974, after 9 years of follow up.

e Only the survival/mortality rate of all inflammatory ocular disorders together is shown, which is comparable to controls in this study.

f Age at onset scleritis.

g Including lung noduli, lung fibrosis, pericardial effusion, vasculitis, polyneuropathy, rheumatoid noduli.

h One patient died due to a pulmonary carcinoma, others from multiple complications due to RA and immunosuppressive therapy.

**Table 3:** Characteristics of current 10 patients with rheumatoid arthritis and scleritis.

Clinical characteristics	Case 1	Case 2	Case 3	Case 4	Case 5	Case 6	Case 7	Case 8	Case 9	Case 10
Age onset RA (y)	43	63	54	59	45	79	52	62	66	24
Age onset Scleritis (y)	56	72	47	58	55	81	60	49	73	46
Duration RA before scleritis (y)	13	9	0	0	10	2	8	0	7	22
Gender (M/F)	M	F	F	F	F	M	F	F	F	F
Race	Cau	Cau	Cau	Asian	Cau	Cau	Cau	Negroid	Cau	Cau
Scleritis first sign of RA	No	No	Yes	Yes	No	No	No	Yes	No	No
Laterality	Bilateral	Bilateral	Bilateral	Bilateral	Unilateral	Unilateral	Bilateral	Bilateral	Unilateral	Bilateral
Scleritis location	Anterior*	Anterior	Pan	Anterior	Anterior	Anterior*	Anterior	Anterior	Anterior	Anterior
Scleritis subtype	Necrosis	Necrosis	Diffuse	Diffuse	Nodular	Diffuse	Necrosis	Nodular	Nodular	Necrosis
Scleral necrosis	Yes	Yes	Yes	Yes	Yes	No	Yes	Yes	Yes	Yes
Adjacent peripheral ulcerative keratitis	No	No	No	No	No	Yes	No	No	No	No
Complications scleritis^a^	Yes	Yes	Yes	No	No	No	No	Yes	No	Yes
Recurrent/chronic scleritis	No	Yes	Yes	Yes	Yes	No	Yes	Yes	Yes	Yes
Previous eye surgery	No	Yes	No	No	No	Yes	No	No	No	No
Other extra-articular manifestations^c^	Yes	Yes	Yes	No	No	Unknown	Yes	No	No	Yes
Erosive RA	Unknown	Unknown	No	No	No	Unknown	Yes	Yes	Unknown	Yes
Biologics received before/during scleritis onset	Abatacept & HCQ for 6 years	MTX for 9 years	NA	NA	Adalimumab for 10years	MTX for 2 years, tapered before onset scleritis	HCQ, sulfasalazine, MTX for 8 years, MTX tapered before onset scleritis	NA	MTX for 17 years	Etanercept & azathioprine for 16, respectively 13 years
IgM RF	POS	NEG	POS	POS	POS	Unknown	POS	POS	POS	POS
IgG anti-CCP	POS	POS	POS	POS	POS	Unknown	POS	POS	POS	POS
IgA RF	POS	NEG	NEG	Unknown	POS	Unknown	POS	NEG	POS	Unknown
IgA anti-CCP	POS	NEG	POS	Unknown	NEG	Unknown	POS	NEG	NEG	Unknown
IgM RA33	NEG	NEG	NEG	Unknown	POS	Unknown	NEG	NEG	NEG	Unknown

RA, Rheumatoid arthritis; DMARD, Disease Modifying Anti-rheumatic Drug; Cau, Caucasian; Pan= Panscleritis; MTX, Methotrexate; HCQ, Hydroxychloroquine; NA, Not applicable*in combination with uveitis anterior.

a Complications of scleritis include cystoid macular edema and papillitis in cases 1 and 10, visual loss in cases 2 and 3, and cataract and glaucoma in case 8.

b Sulfasalazine was administered for three cases of scleritis and was effective in one case and partly effective in two cases. Both abatacept and etanercept was administered in single cases without beneficial effect.

c Other extra-articular manifestations included cutaneous and pulmonary rheumatoid noduli, pericardial effusion, polyneuropat and vasculitis fingertips.

d Sulfasalazine had good effect on RA disease course in one of three patients. Leflonumide had partly an effect in one patient, and etanercept and abatacept had good effect in single patients in which it was administered.

**Figure 1:**
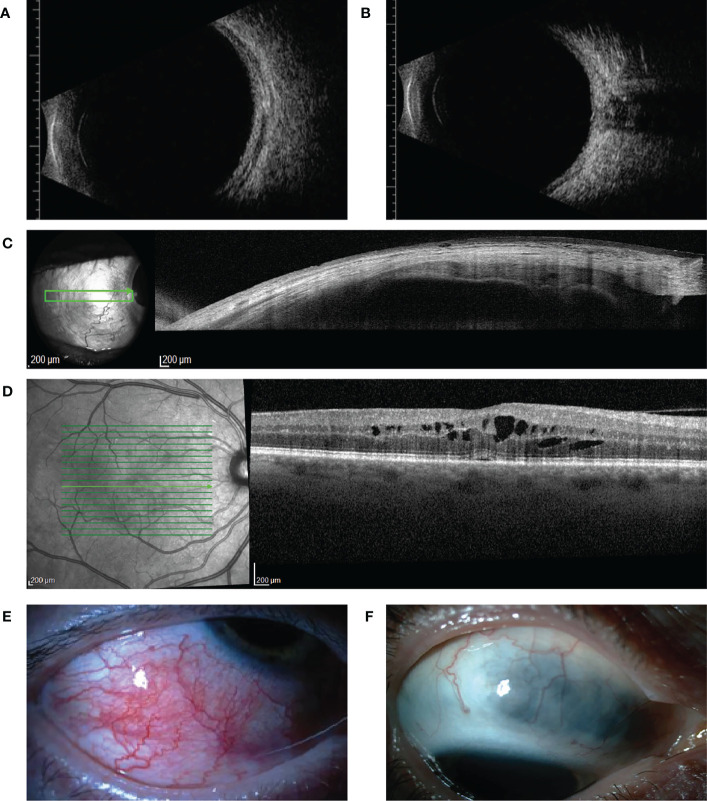
Ultrasound B-scan, optical coherence tomography (OCT) findings, and clinical illustrations of our cases **(A, B)** show ultrasound B-scan images of case 3 with active posterior scleritis. Thickening of the sclera-choroidal complex and fluid accumulation in the episcleral space is seen in **(A, B)** shows fluid accumulation between the optic nerve and the sclera, the for scleritis typical T-sign. **(C)** shows an anterior segment OCT image of case 1, in which thinning of the sclera as a result of scleral necrosis is seen. **(D)** shows cystoid macular edema of case 1, a complication of active scleritis. **(E, F)** show clinical images of respectively case 5 (acute phase of anterior scleritis) and case 10 (quiet stage of previous anterior nodular scleritis following treatment, which exhibits scleral thinning).

Clinical illustrations of our cases are given in **Figure 1**. Clinical characteristics of scleritis described previously are shown in **Table 2**. In earlier decades (before the year 2000), scleritis in patients with RA was characterized by frequent occurrence of complications, specifically scleral necrosis (7, 12, 14, 15). An increased mortality in patients with scleritis and RA was noted and varied between 30-45% (1, 6, 7, 12–14). It was repeatedly observed that patients with scleritis also showed other extra-articular RA manifestations, such as pleuritis, pericarditis and arteritis (12, 14, 15). More recent studies (2018 until now) report on a lower occurrence of scleral necrosis, less severe visual loss, and indicate a decreasing need for surgical intervention (15–17).

### Laboratory findings in patients with scleritis and RA

We found positive IgM-RF in 8/9 scleritis patients (89%) and positive IgG-ACPA in all our patients with scleritis (9/9; 100%). We additionally tested for the other antibody isotypes including IgA-RF and IgA-ACPA, as well as for the presence of IgA/IgG/IgM-RA33 antibodies in the 7 patients with RA of whom stored serum samples were available (**Table 3**). IgA-ACPA was positive in 3/7 patients (43%), and IgA-RF was present in 4/7 patients (57%). More than one ACPA isotype was present in 3/7 patients (43%). IgM-RA33 was present in only 1/7 patients (14%), and IgA/IgG-RA33 was not detected in our cohort. No associations between clinical parameters and the presence of the aforementioned auto-antibodies or their combinations could be established.

## Discussion

Scleritis is one of the severe extra-articular manifestations that can complicate the course of RA. In 1717, William Read described for the first-time ocular disease, later known as scleritis (12). In 1830, Mackenzie attributed scleritis to atmospheric conditions, also known as miasma. He already named it: ‘Scleriotitis atmospherica or rheumatic ophthalmia’. In 1893 the first case of scleral ulceration in a RA patient was described by Holthause (18). In the years thereafter, the relation between RA and (necrotizing) scleritis was well described in several case series, case-control studies, and a few cohort studies (12, 14, 19, 20). Recent clinical observations suggested a decreasing incidence and severity of scleritis in RA, which was attributed to the use of modern therapies, including biologics such as TNFα-inhibitors (3, 21). Our current study shows a cumulative incidence of 0,25% in 10 years, which is indeed to some extent lower than in previous studies.

The disease burden and severity of scleritis in patients with RA were described in detail by Foster et al. in 1984, who reported a high rate of patients with progressive scleromalacia, and a high mortality rate in a cohort before the use of cyclophosphamide (7). Despite the widened current treatment options, our results on scleritis severity are consistent with pre-biologic era reports. The high percentage of patients developing necrosis, even in the current era is striking, though this might have also been influenced by the referral pattern to our ophthalmological tertiary care center.

In our case series, 3 out of 10 patients developed scleritis before the diagnosis of RA was made. This phenomenon was not commonly noted in earlier series, which is probably due to the fact that previous study designs were typically based on inclusion of patients with RA from rheumatologic departments (12, 15, 16). The mean age at onset of RA as well as intervals from RA onset to development of scleritis remained unchanged. Noteworthy is, that all of our patients who developed RA first, had received diverse treatments, including biologics, specifically abatacept, etanercept and adalimumab, before the onset of scleritis, albeit in 3 out of 7 this treatment stopped before scleritis manifested (**Table 3**). This finding indicates that the withdrawal of immunosuppressive therapy may constitute a risk factor for development of scleritis. Interestingly, none of the patients with RA had ever used rituximab before the onset of scleritis.

The recent report on lower incidence of scleral necrosis (51% versus 80% observed by us), found by Caimmi et al. contrasts with our findings and could be explained by the different inclusion criteria. The study by Caimmi et al. includes a rheumatologic population and possibly provides a better representation of overall scleritis severity in RA. The severity of scleritis may be overestimated in studies that included a tertiary ophthalmological population, which was also the case in 3 of our 10 patients (7, 15). Inflammatory parameters in patients with scleritis and RA vary widely between individual studies. In addition, it is not clear at what moment during the course of the disease the samples were taken and evaluated (12, 14). Caimmi et al. reported that the use of immunosuppressive therapy for RA prior to diagnosis of scleritis was common, which could also interfere with the level of inflammatory parameters (16). In our case series, the laboratory assessment was not standardized and took place at different moments during the disease course. In our view, the most informative are data from the active period of the disease prior to any systemic treatment, but such data were (in our series from tertiary center) not available for all. We consider the measurements of general systemic inflammatory markers from not standardized points of time not consistent for evaluation and/or comparison between the specific groups of patients and therefore these were not included in the present series.

Multiple studies noted that scleritis usually occurs in patients with autoantibody positive RA (presence of RF and/or ACPA) (9, 15, 16). We confirm the high prevalence of erosive disease and autoantibody positivity in patients with scleritis and RA (9, 15, 16). Highly specific serological markers for scleritis in RA are not known. Candidate biomarkers for extra-articular involvement in RA were anti-Ro/SSA antibodies (an anti-extractable nuclear antigen antibody; ENA), and a high level of circulating immune complexes (22, 23). The prevalence of IgA-ACPA (43% vs 34% in the general RA population), and IgA-RF (57% vs 51% in the general RA population) in our patients with scleritis and RA did not differ significantly from the general RA population (24). More recently, other RF and ACPA antibody isotypes besides IgM-RF and IgG-ACPA have been tested in RA, as well as the more recently identified anti-RA33 antibody (24). A combination of positive antibodies could better predict the development of RA, and high titers of all isotypes of RF are associated with worse prognosis of RA. It has also been reported that high IgA-RF levels before DMARD initiation are associated with poor treatment response to TNFα-inhibitors (24). In our small sample these antibodies and their relationship with clinical signs of scleritis could not be evaluated.

Limitations of our study include a small number of patients with scleritis and RA, and its retrospective design. Due to the possibility of retrospective study bias we have not included mild ophthalmic problems such as dry eyes in the present study. Further, it would be valuable to study whether a specific treatment modality such a rituximab could protect patients with RA from scleritis development. For this purpose, a large prospective study would be required and a multicenter and/or international collaboration might be necessary.

To conclude, we found a slightly lower incidence of scleritis in the RA population from the last decade compared to the pre-biological era. On the other hand, we were not able to identify a changing clinical pattern of scleritis in the RA population over time. The majority of patients with scleritis and RA developed severe complications and suffered from scleral necrosis. In addition, the mortality of patients with RA affected by scleritis remained high. Rheumatologists and ophthalmologists should remain aware of these severe scleritis symptoms as well as its association with increased mortality.

## Data availability statement

The raw data supporting the conclusions of this article will be made available by the authors, without undue reservation.

## Ethics statement

The studies involving human participants were reviewed and approved by Medical Ethics Review Committee Erasmus MC, Erasmus MC, Rotterdam, the Netherlands.

## Author contributions

Concept and study design: DV, AR, PJ. Data analysis and interpretation: DV, AR, PJ, MS, JT. Drafting the article: DV, AR, PJ. Critical revision of the article: DV, AR, PJ, MS, JT. All Authors contributed to the article and approved the submitted version.
